# The effect of habitat and human disturbance on the spatiotemporal activity of two urban carnivores: The results of an intensive camera trap study

**DOI:** 10.1002/ece3.8746

**Published:** 2022-03-18

**Authors:** Connor Lovell, Shiya Li, Jessica Turner, Chris Carbone

**Affiliations:** ^1^ 4919 Department of Geography University College London London UK; ^2^ Department of Life Sciences Imperial College London Berkshire UK; ^3^ Institute of Zoology Zoological Society of London London UK; ^4^ 4617 School of Biological and Chemical Science Queen Mary University of London London UK

**Keywords:** activity patterns, camera trap, human disturbance, *Meles meles*, urban environments, *Vulpes vulpes*

## Abstract

With rising urbanization, the presence of urban wildlife is becoming more common, increasing the need for wildlife‐friendly spaces in urban planning. Despite this, understanding is limited to how wildlife exploits urban environments and interacts with human populations, and this is vital to our ability to manage and conserve wildlife in urban habitats. Here, we investigate how two urban mammal species, the red fox (*Vulpes vulpes*) and the European badger (*Meles meles*), exploit urban environments. Using intensive camera trap surveys, we assessed how habitat and human disturbance influenced the spatiotemporal activity of these species across south‐west London. Firstly, we found elevated activity levels of both species at boundaries and within built‐up areas, suggesting movement paths follow anthropogenic features. However, badgers were most active in woodland, indicating the importance of high cover habitats suitable for setts and foraging. Secondly, we found badger activity levels were negatively affected by human activity, whilst foxes were unaffected. Further investigation suggested foxes may adapt their activity patterns to avoid human disturbance, with badger activity patterns less plastic. Whilst the results of this study are useful for both the conservation and management of urban wildlife populations, these results also show potential factors which either facilitate or limit wildlife from fully exploiting urban environments.

## Graphical abstract

Using an intensive camera trap study, we identify ecological and anthropogenic factors influencing the spatiotemporal activities of urban badgers and urban foxes. Each species has habitat preferences at two different spatial scales, and urban foxes appear more resilient to human disturbance than urban badgers. In improving our understanding of how these mammals exploit urban environments, future urban conservation and management interventions can be targeted and improved.

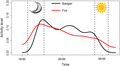

## 1 INTRODUCTION

Since 1960, urban human populations have quadrupled globally, driving the expansion of urban environments (Seto et al., 2010). Frequently characterized by their high human presence and extensively developed land, urban environments present unique challenges for wildlife (Bateman & Fleming, 2012; Gehrt et al., 2010). As urbanization influences animal behavior, and species differ in their ability to exploit urban environments, it is crucial that research is undertaken to elucidate what factors influence the ability of wildlife to thrive in urban environments. However, ecological and anthropogenic factors which influence the activity and distributions of urban wildlife remain unclear (Baker & Harris, 2007).

The European badger (*Meles meles*), a social, sett‐living mustelid, exploits urban environments, either following enclosure by development or active colonization (Baker & Harris, 2007; Bateman & Fleming, 2012; Mathews et al., 2018). Although urban residents value badgers, urbanization harms badger populations through habitat loss and habitat fragmentation and amplifies human‐badger conflicts such as bin raiding, road traffic collisions, and damage to infrastructure from sett excavations (Baker & Harris, 2007; Bateman & Fleming, 2012; Delahay et al., 2009). Habitat has been identified as a factor influencing badger activity; however, previous studies predominantly focus on rural areas, investigating links between badgers and bovine tuberculosis (Balestrieri et al., 2009; Böhm et al., 2018; White et al., 1993). Limited research into the activity of urban badgers has reached conflicting conclusions on habitat preference. In urban landscapes, Cresswell and Harris (1988) detected no clear habitat preferences by badgers. Contrasting this, Davison et al. (2009) detected high preference by badgers for dense scrub, whilst Kauhala and Auttila (2010) demonstrated a preference for sparse undergrowth instead. Rural badgers also avoid habitats situated closer to human settlements (Lara‐Romero et al., 2012; Piza‐Roca et al., 2018). Whilst it is unknown if urban badgers are similarly averse to human disturbance, their response to humans will potentially influence their ability to exploit urban environments.

One mammal known for its ability to exploit urban environments alongside the badger is the red fox (*Vulpes vulpes*; Baker & Harris, 2007; Macdonald et al., 2004; Scott et al., 2014). The red fox is described as one of the most adaptable wild carnivores, with population densities in urban areas exceeding those in rural areas (Bateman & Fleming, 2012; Mathews et al., 2018; Scott et al., 2014). Urban foxes are reported to utilize a wide variety of urban habitats, from underdeveloped areas to city centers, frequently bringing them into contact with humans (Baker & Harris, 2007; Bateman & Fleming, 2012; Cignini & Riga, 1997; Scott et al., 2014; Soulsbury, 2020). Compared to badgers, foxes utilize cities and residential gardens more frequently, and are less dependent on the home den (Baker & Harris, 2007; Bateman & Fleming, 2012; Geiger et al., 2018). Additionally, foxes show some habituation to human presence, an “urban tameness” (Hegglin et al., 2015). These behavioral differences make foxes a useful species to compare with badgers. By investigating how ecological and anthropogenic factors influence the spatiotemporal activity of badger and fox urban populations, we can begin to understand and predict the ability of these species to exploit urban environments, which may inform management and conservation strategies into the future.

Previous investigation of urban badger and fox activity have used telemetry, encompassing both radio‐tracking and the use of GPS collars (Cresswell & Harris, 1988; Davison et al., 2009). Although useful, telemetry has several limitations, as it records only a small subset of the population, has intrinsic biases which favor data from particular habitat types or during certain weather, and potentially influences animal behavior through the tagging and tracking of the individuals in question (Caravaggi et al., 2017; Frair et al., 2010; Tuyttens et al., 2002). Alternatively, following advances in remote sensing technologies, the use of camera traps in ecological studies has increased (Mccallum, 2013). Camera traps can survey continuously and remotely, whilst reducing human interference compared to telemetry, despite the potential for camera traps to be detected by animals and influence behaviors (Meek et al., 2016). Camera trap surveys can therefore complement knowledge gained through telemetry studies by providing a representative, population‐level measure of behavior whilst reducing some of the potential biases resulting from the limited sample sizes involved in telemetry.

In this study, we present the results of an extensive camera trap survey to investigate how habitat and human disturbance influence the spatiotemporal activity of badgers and foxes across an urban landscape in south‐west London. We expect foxes to be more flexible with the habitats they utilize, compared to badgers which we predict to be restricted to high‐cover habitat types such as scrubland and woodland, important habitats for foraging and sett building (Huck et al., 2008; Kruuk, 1978; Zabala et al., 2002). We also predict that fox activity will be more robust to human disturbance than badger activity, based on the greater observed exploitation of urban spaces by foxes, and that badgers will either reduce or shift their activity to avoid human presence, as observed in badger populations subjected to human persecution which are more nocturnal (Bateman & Fleming, 2012; Sidorchuk & Rozhnov, 2018; Tuyttens et al., 2001). Through adding to our understanding of these species’ behavioral responses to urban habitats and human disturbance, this study will increase our understanding of the urbanization potential of these two species and inform future management and conservation strategies.

## 2 METHODS

### 2.1 Survey process

**FIGURE 1:**
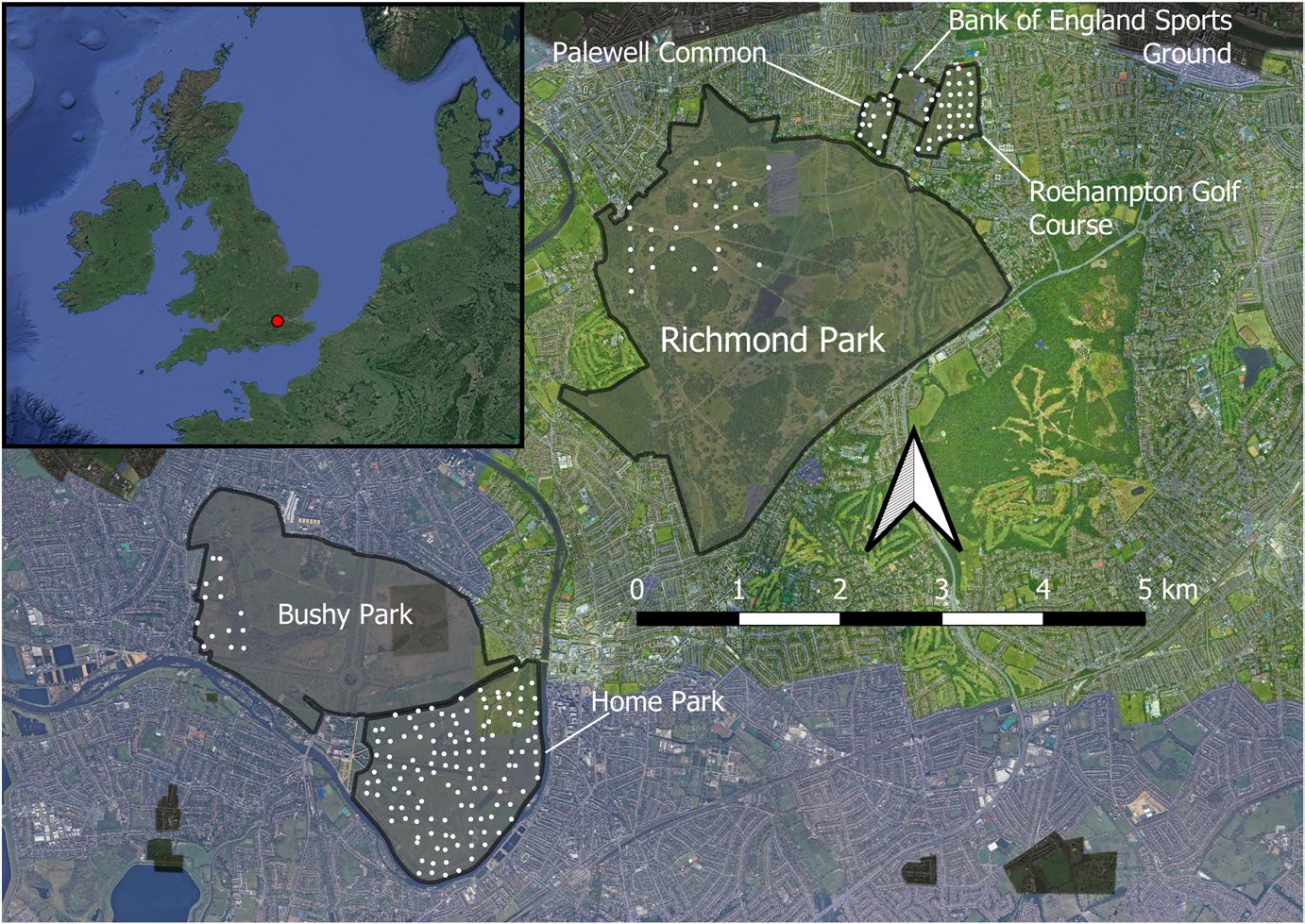
Survey map showing the six camera trap surveys conducted, their locations in south‐west London, and the locations of individual camera traps. The insert visualizes the location of the survey sites within the UK

Six camera trap surveys, totaling 211 camera traps, were conducted by London HogWatch volunteers across August and September from 2017 to 2019, encompassing approximately 4.75 km^2^ across south‐west London, as part of the Zoological Society of London’s (ZSL) long‐term camera trap studies across London’s landscapes (Figure 1). For each survey, we generated camera trap coordinates using random systematic sampling. From an initial, random coordinate, we generated a 150 m^2^ grid and overlaid this across the survey site, and subsequently positioned cameras at gridline intersections or to the nearest solid fixture. The minimum distance between cameras was 50.11 m, and the maximum distance 9528 m. We attached cameras 20–50 cm high and angled them to maximize the field of view toward the initial gridline intersection. We used four camera trap models: The Browning Strike Force HD Pro, the Reconyx HyperFire HC 500, the Reconyx HyperFire HC 600, and the Bushnell Core™ No Glow Trail Camera. All cameras were configured with a 1 s delay time and used infra‐red flashes to photograph target species at night. From prior work, we did not anticipate significant differences in detection probability for the species between camera trap models, as the detection range for both badgers (approximately 2.5 m) and foxes (approximately 3.5 m) lies within the total detection range of all camera trap brands used (Ravera & Carbone, 2019). All brands also have a trigger speed of <0.3 s.

### 2.2 Image tagging and data extraction

Once cameras were recovered, we downloaded and manually tagged images with any badgers or foxes present using *ExifPro* v10.3.3.0.1 (Kowalski, 2013). Each camera captured an image sequence of an animal, with a 1 s delay between images, until that animal left the field of view. Only the first image of each sequence, marked as a “contact,” was used in future analyses. Only images captured between 18:00 and 08:00 were tagged, as the target wildlife species are typically nocturnal and daytime recordings are inflated by non‐target animals, which lead to a high number of total photographs (Mori et al., 2020). This ensured all cameras were tagged for at least 1 h after sunrise, and the vast majority were tagged for at least 1 h before sunset. We anticipated this practice to have minimal impact on the results.

### 2.3 Habitat classification

Camera trap locations were plotted onto a Google Earth satellite image using QGIS v2.18.24 (QGIS Development Team, 2020).

We assigned two categorical habitat classifications to each camera site, representing different landscape scales, which we developed based on Phase 1 Habitat Survey classifications (Joint Nature Conservation Committee, 2010). Habitats were assigned remotely based on Google Earth satellite imagery and street view (Google, 2020), camera trap photographs, and Digimap habitat class data (EDINA, 2020). First, we assigned a point habitat to each camera site, representing the immediate surrounding habitat. Next, we added a 50 m buffer around each camera and, dictated by the habitat which encompassed the largest area within this buffer, assigned a second habitat class (Table 1; Niedballa et al., 2015; Ordenana et al., 2010). Fifty meter was used as a higher selection order, whilst additionally being a reasonable distance to assess the wider habitat characteristics which could influence the camera trap site (Johnson, 1980).

**TABLE 1:** The dichotomous keys used to assign point and 50 m habitat classifications. For each camera trap, starting at (1) each key was followed and used to assign two habitat classifications

*Point habitat classification key*
This habitat class applied to the habitat in direct proximity and immediately surrounding each camera trap
1. Is the camera trap located within or in direct proximity to any anthropogenic features, such as roads with an impervious surface, buildings, gardens, fences, or walls? Yes = go to 2 | No = go to 3 2. Is the camera trap located directly adjacent to a road, defined as an impervious surface which vehicles could utilize? Yes = Road Verges | No = go to 4 3. Is the camera trap located within trees or shrubs, regardless of number? Yes = go to 5 | No = Amenity Grassland 4. Is the camera trap located alongside and in direct proximity to a fence or wall? Yes = Boundaries | No = Built**‐**up Environments 5. Is the camera trap located within an area dominated by vegetation consisting primarily of shrubs and scrubland, as opposed to trees? Yes = Scrubland | No = go to 6 6. Is the camera trap located within trees which form a discontinuous canopy, or are few in number and isolated within a more open landscape? Yes = Scattered Trees | No = Woodland
*50 m habitat classification key*
This habitat class applied to the dominant habitat class, defined as the habitat class which encompasses the largest area within a 50 m buffer, around each camera trap
1. Does the dominant habitat consist of anthropogenic features, such as roads with an impervious surface, buildings, or gardens? Yes = Built**‐**up Environments | No = go to 2 2. Does the dominant habitat contain trees or shrubs, regardless of number? Yes = go to 3| No = Amenity Grassland 3. Does the dominant habitat consist primarily of shrubs and scrubland, as opposed to trees? Yes = Scrubland | No = go to 4 4. Does the dominant habitat consist of trees which form a discontinuous canopy, or are few in number and isolated within a more open landscape? Yes = Scattered Trees | No = Woodland

### 2.4 Quantifying human disturbance

We tagged human contacts over the full 24‐h period at Home Park using a classifier trained on known human images. It is important to consider the full 24‐h cycle of human activity, despite both target animal species being primarily nocturnal, because we are interested in the full impact of human activity on the species—including indirect effects from diurnal human activity. This classifier first used the pretrained Convolutional Neural Network *Inception*‐*v3* to extract features from images, before training with and using a Multi‐layer Perceptron algorithm to identify images with humans present (Rumelhart et al., 2019; Szegedy et al., 2015). As above, the first image of a sequence of human images was defined as a “contact,” with a new sequence beginning once at least 1 min had passed between human images.

To estimate human disturbance, we used the number of human contacts per day at each camera trap (the human contact rate). Only Home Park was utilized for this analysis because of limitations in the ability of the classifier to accurately classify humans at other parks. As a result, only wildlife contacts from Home Park were utilized in this analysis investigating the influence of human activity.

### 2.5 Statistical analyses

Statistical analyses were performed with R v4.0.0 (R Core Team, 2020). Unless stated, we used *activity* v1.3 (Rowcliffe, 2019) for activity pattern estimation and analysis. Figures were created by R, *jtools v2*.*1*.*3* (Long, 2020) and *ggplot2* v3.2.1 (Wickham, 2016). Tables were created by *sjPlot* v2.8.4 (Ludecke, 2020). Results were deemed significant if |Z statistic| > 2 or if *p*‐value < .05 (Luke, 2017).

### 2.6 Detection rates by habitat

We first investigated the influence of habitat on detection rates. Using *lme4* v1.1 (Bates et al., 2015) and *MASS* v7.52 (Ripley et al., 2020), we modeled badger and fox activity across cameras using negative binomial generalized linear mixed models (GLMM), as we detected overdispersion via *performance* v0.46.6 (Dispersion ratios for all the following models ranged from 9.64–27.42, with *p*‐values all < .001, Ludecke et al., 2020).

Following Sollmann (2018), the detection rate of each animal acted as a proxy for that animal’s activity at a given camera trap, averaged over time. Therefore, we utilized the *number of badger contacts* or *number of fox contacts* as the response variable, with the *log of the trapping effort* (the number of days each camera trap was deployed for) as an offset variable. This offset variable accounted for varying deployment times across cameras, and ensured the response variable acted as a detection rate (Sollmann, 2018). We used either the *point* or *50 m habitat* as the explanatory variable. This led to four different GLMMs: two for modeling badger activity with point and 50 m habitat, and two for modeling fox activity with point and 50 m habitat, each with only one explanatory variable being tested.

We included the *survey location* as a random factor, to account for spatial autocorrelation (Dormann et al., 2007). Survey locations used as random factors were Bushy Park, Home Park, Richmond Park, and the Roehampton area (consisting of the Bank of England Sports Ground, Palewell Common, and Roehampton Golf Course). The former three parks are surrounded by high brick walls, severely restricting badger movement between them, whilst the Roehampton area likely permits badgers to move between the three survey sites. *R* ^2^ were calculated to assist in interpretation of the model results. Following model construction, we computed *post hoc* pairwise comparisons of estimated marginal means between habitat classes via *emmeans* v1.4.7 (Lenth, 2020).

### 2.7 Activity patterns of badgers and foxes

To estimate badger and fox activity patterns, we used non‐parametric circular kernel density estimation. Using individual contact times converted to radian time‐of‐day, kernel density estimation produced a continuous probability density function representing each species’ activity pattern (Ridout & Linkie, 2009). We then compared badger and fox activity patterns via *circular* v0.93 (Agnostinelli & Lund, 2017), using Watson–Wheeler tests of circular homogeneity. The Watson–Wheeler test is a non‐parametric test to test whether two samples of cyclic data differ significantly.

### 2.8 Responses to human activity

To investigate the influence of human detection rates on badger and fox detection rates, we modeled badger and fox activity using negative binomial GLMs due to overdispersion, with the *number of badger contacts* or *number of fox contacts* as the response variable. The *human detection rate* was the explanatory variable, with the *log of the trapping effort* used as an offset variable.

Finally, to investigate the influence of human disturbance on badger and fox activity patterns, we ranked camera traps by their human contact rate to identify the sites most and least disturbed by humans, before isolating badger and fox records associated with the lower and upper third of camera traps, thereby corresponding with the lowest and highest recorded human activity levels. These subsets of records were then used to construct corresponding activity patterns as described previously. Similarly, badger and fox activity patterns from the most and least disturbed sites were compared using Watson–Wheeler tests of circular homogeneity.

## 3 RESULTS

Species recordings were obtained from 211 camera trap placements between August and September in 2017, 2018, and 2019. Camera traps were deployed for an average of 13.19 nights (ranging from 1 to 32 nights), with a total of 2784 camera trap nights. Naïve occupancy, defined as the proportion of sites where each target species was detected, was 0.49 for badgers and 0.71 for foxes. On average, it took one night to detect fox presence, and three nights to detect badger presence, suggesting that a shorter trapping period didn’t significantly bias results. Throughout the surveys, 933 independent badger contacts and 4226 independent fox contacts were recorded, with Home Park recording 497 independent badger contacts and 489 independent fox contacts.

### 3.1 Detection rates by habitat

At the point scale, 19 camera traps were located in amenity grassland, 44 along boundaries, 2 in built‐up areas adjacent to buildings and other anthropogenic structures, 15 along road verges, 91 in scattered trees, 12 in scrubland, and 28 in woodland. At the 50 m scale, 134 camera traps were located in amenity grassland, 10 in built‐up areas, 31 in scattered trees, 4 in scrubland, and 32 in woodland.

At the point habitat scale, the GLMM predicted badger detection rates to not differ significantly from the intercept of amenity grassland in most habitats. Badger detection rates were significantly elevated at boundaries and in woodland, recording one badger per 5 days at boundaries (GLMM; CI 2.18–24.26, Z_211_ = 3.23) and one badger per 2 days in woodland (GLMM; CI 3.90–49.86, Z_211_ = 4.05). Scrubland almost demonstrated significantly elevated badger activity, at one badger per 7 days (GLMM; CI 0.10–21.86, Z_211_ = 1.96, Table 2). Additional pairwise comparisons detected elevated detection rates in woodland compared to road verges (GLMM; estimate = −1.98, SE = 0.60, Z_211_ = −3.32) and scattered trees (GLMM; estimate = −1.64, SE = 0.38, Z_211_ = −4.37), and at boundaries compared to scattered trees (GLMM; estimate = 0.99, SE = 0.33, Z_211_ = 3.02).

**TABLE 2:** GLMM outputs from testing the effect of habitat on badger activity at both habitat scales

Explanatory variables	Point habitat			50‐meter habitat		
	Incidence Rate Ratios	95% Confidence Interval	Z test statistic	Incidence Rate Ratios	95% Confidence Interval	Z test statistic
Amenity Grassland (Intercept)	0.03	0.01–0.19	**−3.74**	0.09	0.02–0.30	**−3.72**
Boundaries	7.27	2.18–24.26	**3.23**	–	–	–
Built‐up	4.11	0.29–58.72	1.04	4.86	1.50–15.76	**2.63**
Road Verges	1.92	0.42–8.84	0.84	–	–	–
Scattered Trees	2.71	0.84–8.71	1.67	1.79	0.88–3.63	1.61
Scrubland	4.67	0.10–21.86	1.96	1.29	0.22–7.42	0.28
Woodland	13.94	3.90–49.86	**4.05**	5.41	2.76–10.60	**4.92**
Random effects						
σ^2^	1.42	1.42				
τ_00_	2.09_location_	1.59_location_				
ICC	0.60	0.53				
*N*	4_location_	4_location_				
Observations	211	211				
Marginal *R* ^2^ / Conditional *R* ^2^	.14 / .65	.12 / .59				
AIC	835.45	834.02				

Note

Bold indicates statistical significance, where |Z| > 2. Except for the intercept of amenity grassland, the incident rate ratios represent the multiplicative change in contact rate attributable to each explanatory variable. The intercept of amenity grassland representing the number of contacts in that habitat per day. The confidence intervals then represent the 95% confidence interval for this value.

At the 50 m habitat scale, the GLMM predicted significantly elevated badger detection rates in built‐up environments and woodland, recording one badger per 2 days in built‐up environments (GLMM; CI 1.50–15.76, Z_211_ = 2.63), and one badger per 2 days in woodland (GLMM; CI 2.76–10.60, Z_211_ = 4.92). Additionally, pairwise comparisons detected elevated detection rates in woodland compared to scattered trees (GLMM; estimate = −1.11, SE = 0.45, Z_211_ = −2.48).

At the point habitat scale, the GLMM predicted little habitat preference by foxes compared to the intercept of amenity grassland; only an avoidance effect was detected in scattered trees (GLMM; CI 0.23–0.95, Z_211_ = −2.10; Table 3). However, pairwise comparisons detected elevated detection rates at boundaries compared to road verges (GLMM; estimate = 1.27, SE = 0.41, Z_211_ = 3.10), scattered trees (GLMM; estimate = 1.48, SE = 0.24, Z_211_ = 6.29), scrubland (GLMM; estimate = 1.27, SE = 0.44, Z_211_ = 2.91), and woodland (GLMM; estimate = 0.97, SE = 0.31, Z_211_ = 3.18).

**TABLE 3:** GLMM outputs from testing the effect of habitat on fox activity at both habitat scales

Explanatory variables	Point habitat			50‐meter habitat		
	Incidence rate ratios	95% Confidence Interval	Z test statistic	Incidence rate ratios	95% Confidence Interval	Z test statistic
Amenity Grassland (Intercept)	0.83	0.32–2.13	−0.39	0.54	0.29–1.01	−1.94
Boundaries	2.08	0.10–4.35	1.95	–	–	–
Built‐up	1.31	0.21–8.17	0.29	6.01	2.48–14.54	**3.98**
Road Verges	0.58	0.22–1.51	−1.11	–	–	–
Scattered Trees	0.47	0.23–0.95	**−2.10**	1.24	0.72–2.15	0.78
Scrubland	0.59	0.22–1.56	−1.07	0.87	0.22–3.53	−0.19
Woodland	0.79	0.36–1.74	−0.59	1.50	0.89–2.55	1.52
Random effects						
σ^2^	0.91	0.99				
τ_00_	0.51_location_	0.33_location_				
ICC	0.36	0.25				
*N*	4_location_	4_location_				
Observations	211	211				
Marginal *R* ^2^ / Conditional *R* ^2^	.19 / .48	.11 / .33				
AIC	1190.58	1206.69				

Note

Bold indicates statistical significance, where |Z| > 2. Except for the intercept of amenity grassland, the incident rate ratios represent the multiplicative change in contact rate attributable to each explanatory variable. The intercept of amenity grassland representing the number of contacts in that habitat per day. The confidence intervals then represent the 95% confidence interval for this value.

At the 50 m habitat scale, the GLMM again predicted little habitat preference by foxes compared to the intercept of amenity grassland. The exception was built‐up environments, which recorded an elevated fox detection rate at three foxes per day (GLMM; CI 2.48–14.54, Z_211_ = 3.98). Furthermore, pairwise comparisons found activity was elevated in built‐up environments compared to scattered trees (GLMM; estimate = 1.58, SE = 0.51, Z_211_ = 3.10) and woodland (GLMM; estimate = 1.39, SE = 0.49, Z_211_ = 2.84).

### 3.2 Activity patterns of badgers and foxes

**FIGURE 2:**
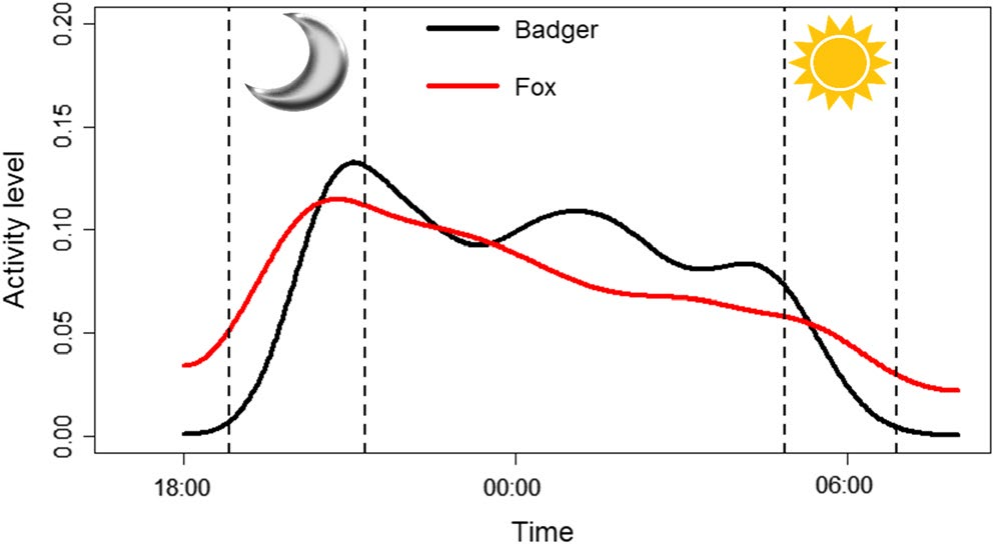
Activity patterns of badgers (black) and foxes (red) averaged over all camera traps throughout the night. Nine hundred thirty‐three independent badger contacts and 4226 independent fox contacts were used. Kernel density on the y‐axis acts as a proxy for activity level at a given time. Dotted vertical lines represent the sunset (moon) and sunrise (sun) time periods over the course of the survey seasons

Badger and fox activity patterns differed significantly from each other (W_2_ = 59.36, *p* < .001). Fox activity appeared higher earlier in the evening and later in the morning, whilst badger activity was higher throughout the middle of the night (Figure 2).

### 3.3 Responses to human activity

**FIGURE 3:**
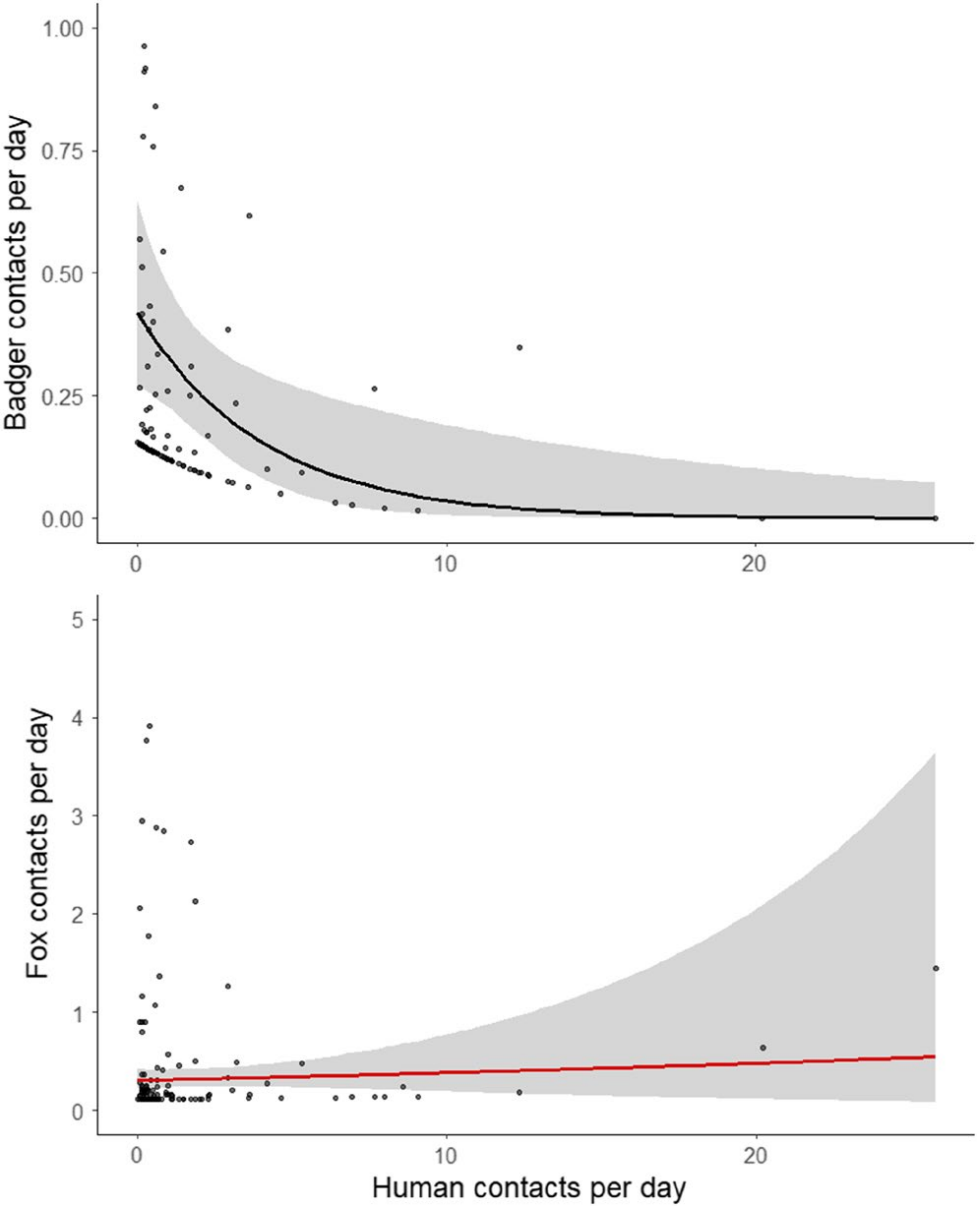
Poisson regressions plotted from modeling badger (black) and fox (red) activity as a function of human activity. Points represent partial residuals to account for varying camera trap deployment times. Hence, values < 1 represent where a mammal contact has been registered less than once per day (i.e., 0.50 represents 1 contact per 2 days). Shading represents 95% confidence intervals

When human activity increased at camera sites by one human per day, the GLM predicted badger activity to decrease by 22% (GLM; estimate = −0.247, SE = 0.094, Z_125_ = −2.621, *p* = .009), whereas fox activity was not significantly affected (GLM; estimate = 0.023, SE = 0.039, Z_125_ = 0.774, *p* = .566, Figure 3).

**FIGURE 4:**
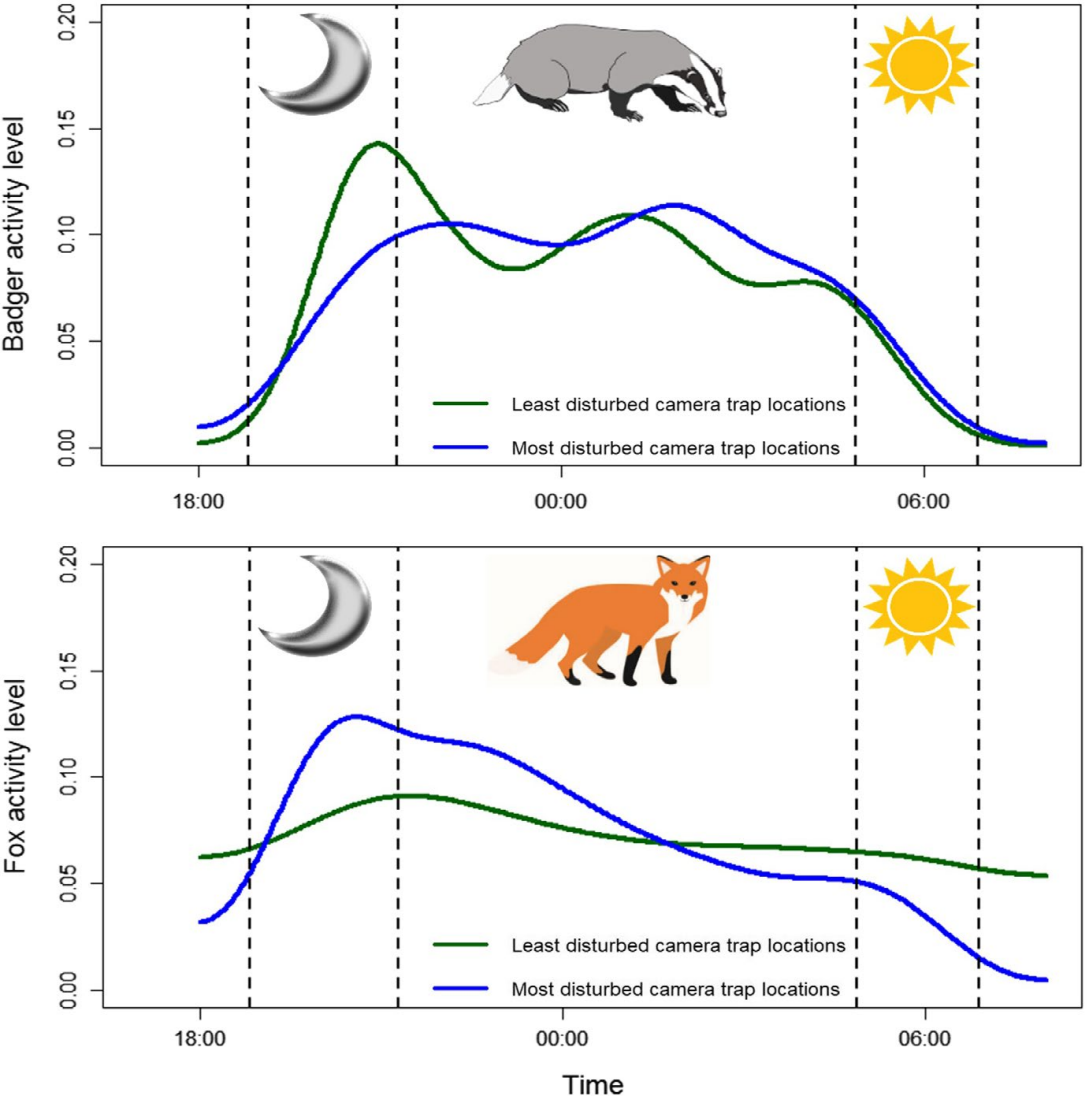
Activity patterns of badgers and foxes at the least (green) and most (blue) disturbed camera traps. At the least disturbed camera traps, 329 badgers and 115 foxes were observed. At the most disturbed camera traps, 63 badgers and 253 foxes were observed. Kernel density on the y‐axis acts as a proxy for activity level at a given time. Dotted vertical lines represent the sunset (moon) and sunrise (sun) time periods over the course of the survey seasons

When comparing activity patterns of the most disturbed camera traps with the least, badger activity patterns did not differ significantly (Watson–Wheeler test; W_2_ = 1.78, *p* = .410). Fox activity patterns were not significantly different at the significance threshold; however, they were only marginally insignificant (Watson–Wheeler test; W_2_ = 5.90, *p* = .052, Figure 4).

## 4 DISCUSSION

This study investigated how habitat and human activity influenced the spatiotemporal activities of urban badgers and urban foxes. Our results suggest both urban badgers and urban foxes have differing habitat preferences occurring at different spatial scales, although model fit was low meaning the results should be interpreted with caution. Furthermore, urban badgers appeared negatively affected by human presence, unlike the fox, which were more plastic in their activity patterns.

Badger activity was elevated in woodland at both spatial scales. Here, badger activity may be elevated because setts are likely to be located in woodland, benefiting from high cover and the structural support of root systems (Feore & Montgomery, 1999; Huck et al., 2008; Piza‐Roca et al., 2018). Additionally, badgers are omnivorous and spend a large proportion of their time foraging for earthworms, including within woodland environments (Kruuk, 1978; Mathews et al., 2018; Zabala et al., 2002). Furthermore, the cover provided by woodland may be selected by badgers moving through the environment as light levels increase with sunrise (Davison et al., 2009; Piza‐Roca et al., 2018). Therefore, woodland appears to remain an important habitat for urban badgers.

In addition to woodland, badger activity was also elevated along boundaries, representing linear structures such as fences and walls. One potential reason for this is that the impermeable nature of some of these boundaries, such as walls, could be forcing badgers to move alongside them, thereby inflating camera trap detection rates. However, pairwise comparisons of habitat use for foxes also show elevated activity at boundaries, compared to road verges, scattered trees, scrubland, and woodland habitats, even though they are unlikely to represent such a strong barrier for this species. Therefore, an alternative explanation is that these species may preferentially move along boundary features. Indeed, previous studies have identified that badger latrines and urinations are preferentially positioned alongside boundary features, which are hypothesized to channel badger movements and result in a higher likelihood of information transfer via scent marks in scat and urine (Balestrieri et al., 2009; Stewart et al., 2002). A similar channeling effect could explain the observed elevated fox activity along boundaries compared to most other habitat types, as foxes also use boundary features, such as fences and walls, to deposit scent marks (Baker et al., 2000). This act of moving along boundary features (previously named linear feature tracking) has been identified in rural populations of foxes via GPS, whilst research into rural badgers also indicates higher activity levels at more permeable boundary features such as woodland edges and ditches (Bischof et al., 2019; Stewart et al., 2002; White et al., 1993). Boundaries may therefore play an important ecological role by facilitating communication and commuting behavior in both species in urban environments, though further research is needed to understand how.

Both badgers and foxes showed increased activity in built‐up environments, defined as being in proximity to anthropogenic features such as roads, houses, and gardens, when measured at the 50 m scale. Foxes are well documented to exploit urban landscapes and residential areas for supplemental anthropogenic food resources, shelter, and breeding (Bateman & Fleming, 2012; Contesse et al., 2004; Harris, 1981). However, it is less clear why badger activity is elevated in built‐up environments. For both foxes and badgers, these results could be an artificial effect of badgers and foxes using boundaries, which are often located alongside built‐up areas. However, urban badgers could also be showing some ability to utilize built‐up areas, contrasting Lara‐Romero et al. (2012) and Piza‐Roca et al. (2018) who suggest rural badgers avoid habitats near human settlements. Indeed, Davison et al. (2009) recorded selection for, and slow travel speed through, gardens by urban badgers, likely reflecting a tendency for gardens to be used as foraging areas. Furthermore, Harris (1984) demonstrated urban badgers obtain up to 42% of their diet from scavenged anthropogenic food. This provides some evidence toward potential behavioral differences between urban and rural badgers, with urban badgers able to better exploit anthropogenic environments.

This study found that both species appear able to exploit built‐up areas. However, unlike foxes, badgers were also found to respond negatively to human activity, with a predicted 22% decrease in badger activity for every extra human per day recorded. This suggests a contrast between badgers both utilizing built‐up areas whilst also being less able to tolerate human activity. This differential response to development and actual human presence has previously been identified in North American mammalian fauna. Nickel et al. (2020) demonstrated that smaller mammalian predators avoid areas of high human activity whilst also preferring developed areas, whereas Suraci et al. (2021) demonstrated how development and human activity influence occupancy rates differentially of several mammal species across trophic levels. This study supports these findings with a similar pattern observed in badgers and indicates the need for future research to untangle the effects of development footprint and human activity. With regards to urban badgers, human activity specifically could therefore be limiting the ability of badgers to move through more built‐up areas, inhibiting dispersal. Indeed, badgers in urban areas are less likely to utilize outlier setts, instead spending more time at one main sett (Davison et al., 2008). However, it is also possible that other anthropogenic pressures not investigated in this study, such as light or noise, could also be influencing this apparent contrasting response of badgers to development and human behavior.

Underpinning the differential responses of badgers and foxes to human detection rates could be the relatively inflexible badger activity pattern, compared to the more plastic fox activity pattern. Whilst marginally insignificant, the activity pattern of foxes varied more between the most and least disturbed camera traps. This could suggest foxes utilizing more disturbed camera trap sites adopt a more nocturnal activity pattern, potentially to avoid human activity. This is supported by previous research, with urban foxes in Sydney more nocturnal than their peri‐urban counterparts, indicating activity pattern plasticity (Gil‐Fernández et al., 2020). Foxes could therefore be able to adapt their activity patterns to increasing anthropogenic disturbance, allowing them to exploit areas with high human activity. Contrasting foxes, badgers show a similar activity pattern at both the least and most disturbed camera traps. The reduction in badger activity at highly disturbed sites could stem from this less plastic activity pattern, meaning badgers fail to avoid humans to a suitable level and instead avoid these sites. Further research is needed to identify the reason why badgers are more susceptible to human disturbance.

In this study, detection rate was utilized as a proxy for activity. Although this is a commonly used proxy for overall activity levels of a species at a camera trap site, limitations do remain (Sollmann, 2018). Firstly, it is unknown whether a higher activity level reflects one individual visiting the site multiple times over the course of the trapping period, or multiple individuals of one species. Additionally, it is unknown whether certain covariates of camera trap sites are likely to bias detection. For example, a camera trap positioned in amenity grassland may be more likely to be triggered by a passing individual than a camera trap in scrubland, where vegetation obscures much of the view. Furthermore, badgers and foxes shift their activity patterns seasonally, meaning these results are only applicable to August and September (Torretta et al., 2016). Finally, should humans notice more visible camera traps they may avoid, or indeed attempt to trigger, camera traps, which could risk biasing estimates of human activity.

With human urban populations predicted to increase to six billion by 2044, these results have important consequences for our understanding of how badgers and foxes may react to the future expansion of urban areas, informing future urban management and conservation programs (Seto et al., 2010). Whilst these results find that foxes appear to be adaptable to urban environments, this study identified two key factors which could limit badgers from exploiting urban areas: a lack of woodland and human disturbance. As expansion of woodland is difficult in an urban setting, conservation of remaining urban woodland will likely benefit badger populations, alongside other wildlife (Croci et al., 2008). In addition, although badgers appear able to traverse built‐up areas, consideration must be given to elevated human activity within these areas, which risks limiting badgers dispersal, as well as impairing important behaviors such as sett maintenance (Tuyttens et al., 2001).

The utilization of intensive camera trap surveys in this study provided a unique and detailed insight into the spatiotemporal activity of urban badgers and foxes, and how they are influenced by urban habitats and human disturbance. Future research should further investigate and compare the activities of these two species, by combining the camera trapping utilized here with methods such as telemetry and citizen science (Bischof et al., 2019; Davison et al., 2008; Harris, 1981). Additionally, this study focusses on two contrasting carnivores’ responses to habitat and human activity, but future studies could investigate interactions between the species and other mammals in the urban environment. Further untangling of the impact of development and human activity on mammals, alongside other potential forms of disturbance, will ensure future urban conservation and management interventions can be targeted and improved.

## CONFLICT OF INTEREST

The authors declare no conflicts of interest.

## AUTHOR CONTRIBUTIONS

**Connor Lovell:** Conceptualization (lead); Data curation (equal); Formal analysis (equal); Methodology (equal); Writing – original draft (lead); Writing – review & editing (equal). **Shiya Li:** Formal analysis (equal); Writing – review & editing (equal). **Jessica Turner:** Conceptualization (supporting); Supervision (supporting); Writing – review & editing (equal). **Chris Carbone:** Conceptualization (supporting); Data curation (equal); Methodology (equal); Resources (lead); Supervision (lead); Writing – review & editing (equal).

## Data Availability

The dataset from this study will not be made publicly available online as it contains information on the locations of a sensitive and protected species (the European badger; *Meles meles*).
